# Juxtaposing Medical Centers Using Different Questionnaires Through Score Predictors

**DOI:** 10.3389/fnins.2022.818686

**Published:** 2022-03-23

**Authors:** Clara Puga, Miro Schleicher, Uli Niemann, Vishnu Unnikrishnan, Benjamin Boecking, Petra Brueggemann, Jorge Simoes, Berthold Langguth, Winfried Schlee, Birgit Mazurek, Myra Spiliopoulou

**Affiliations:** ^1^Knowledge Management & Discovery Lab, Otto-von-Guericke University Magdeburg, Magdeburg, Germany; ^2^Tinnitus Center, Charité-Universitaetsmedizin Berlin, Corporate Member of Freie Universität Berlin and Humboldt-Universität zu Berlin, Berlin, Germany; ^3^Department of Psychiatry and Psychotherapy, University of Regensburg, Regensburg, Germany

**Keywords:** tinnitus, networks, similarity, socio-demographics, adherence, predictive modeling

## Abstract

**Background:**

Chronic tinnitus is a clinically multidimensional phenomenon that entails audiological, psychological and somatosensory components. Previous research has demonstrated age and female gender as potential risk factors, although studies to this regard are heterogeneous. Moreover, whilst recent research has begun to identify clinical “phenotypes,” little is known about differences in patient population profiles at geographically separated and specialized treatment centers. Identifying such differences might prevent potential biases in joint randomized controlled trials (RCTs) and allow for population-specific treatment adaptations.

**Method:**

Two German tinnitus treatment centers were compared regarding pre-treatment data distributions of their patient population bases. To identify overlapping as well as center-specific factors, juxtaposition-, similarity-, and meta-data-based methods were applied.

**Results:**

Between centers, significant differences emerged. One center demonstrated some predictive power of the patients of the other center with regard to questionnaire score after treatment, indicating similarities in treatment response across center populations. Furthermore, adherence to the completion of the questionnaires was found to be an important factor in predicting post-treatment data.

**Discussion:**

Differential age and gender distributions per center should be considered as regards RCT design and individualized treatment planning.

## 1. Introduction

Tinnitus can be described as a phantom auditory perception (Jastreboff, 1990). When investigating tinnitus prevalence, age and gender are commonly analysed variables. While age is considered to be an important factor in predicting tinnitus severity, the effect of gender does not reach consensus yet among the tinnitus research community (Biswas and Hall, 2020). There is also no conclusive evidence linking tinnitus or patient characteristics to treatment outcome (Schlee et al., 2021).

The goal of this study is to expand on previous research by investigating not only tinnitus patient characteristics within a clinical center, but also whether patients from one center are predictive of patient post-treatment data from another center. Therefore, we investigate how transferable the knowledge gathered in one center is to the other. In summary, the following research questions are tackled:

**RQ1:** To what extent do age and gender distributions within clinical centers reflect the age and gender distributions of the general population?
**RQ2:** How are the similarities and differences in both centers with respect to age, gender, and questionnaire scores?
**RQ3:** To what extent are pre-treatment data of one center predictive of post-treatment data of patients from the same center and from the other center? Does gender improve post-treatment data predictions?

To investigate the interplay between the age variables from the general German population and the tinnitus patients (RQ1), we firstly use a method to juxtapose the two distributions (age in Germany and age in the center) using a kernel density estimation plot. This method helps highlighting the parts of the distributions that are very similar and those which are not. We also analyse the differences in age distributions per gender and per center. As a result, the age distribution of one center's female tinnitus patients is compared to the age distribution of the other center's female tinnitus patients. The same reasoning is used to the male tinnitus patients. Gender is also compared between centers and with the distribution in Germany.

Our approach to uncover similarities in distributions of questionnaire scores by gender and clinical center (RQ2) is network-based. We design the network with nodes as patients and edges as a distance function that measures the difference between the questionnaire scores of each pair of patients. We use the same representation as in a recent work (Puga et al., 2021). After mapping the data into a network representation, we use the netLSD distance to compute and compare the distance across networks. These distances can then be rated, with shorter distances suggesting higher similarity between patients.

Finally, we train models to predict post-treatment questionnaire data (i) within each center and then (ii) with the other center's data (RQ3). These models are computed with the complete sample and per gender. There are two main goals for such analysis: (i) to detect if a center is predictive of the other and (ii) to detect if same-gender patients from one center are predictive of the same-gender patients of the other center. The adherence behavior of the patients to filling out the questionnaires is included as different sets of features in the models. Hence, the extent to which these adherence features improve the prediction of the post-treatment score is also investigated.

The remainder of this article is organized as follows: section 2 presents the dataset properties used for the analyses, section 3 describes the methodology to find the relationship of age and gender with tinnitus prevalence organized per research question, section 4 shows the results of our methods to respond to each research question. In section 5 the results are discussed with respect to the available literature and section 6 summarizes the main findings.

## 2. Materials

The data used in this study are from patients with chronic tinnitus who were admitted to the University Hospital of Regensburg and to the Tinnitus Center, Charité - Universitätsmedizin Berlin. The Regensburg University Hospital collected the data between January 3, 2016 and May 28, 2020. The data from the Tinnitus Center, Charité - Universitätsmedizin Berlin were gathered between January 1, 2011 and October 15, 2015. Despite the fact that both datasets contain data from many questionnaires and socio-demographic data, some variables appear only in one dataset and vice versa. Table 1 shows the number of patients with available data at different time points as well as the number of different treatments that were assigned. Two time points were considered: *t*_0_ and *t*_1_. The former denotes the time point at admission, while the latter represents the time at the final visit of the patient to the clinical center. For the sake of simplicity, University Hospital of Regensburg is denoted by UHREG and the Tinnitus Center, Charité - Universitätsmedizin Berlin is denoted by CHA.

**Table 1:** Number of patients and treatment with no missing values at *t*_0_, *t*_1_ and *t*_0_ and *t*_1_.

**Center**	**Time**	**No. of patients**		**No. of treatments**

		**f**	**m**	
UHREG	*t*_0_	260	473	19
	*t*_1_	51	108	16
	*t*_0_ and *t*_1_	24	46	9
CHA	*t*_0_	1,828	1,994	1
	*t*_1_	916	885	1
	*t*_0_ and *t*_1_^*^	852	807	1

* *500 randomly selected patients were used for the analysis*.

Specific data pre-processing steps are required depending on the type of analysis performed. The three research questions require different filters. I.e., for RQ1 we consider all patients from both centers who filled out the age and gender question among all questionnaires, at admission. RQ2 and RQ3 require a match on the questionnaire data that are being compared. This phase is critical for the juxtaposition of the medical centers' questionnaires and ensuring the feasibility of the comparison.

Table 2 summarizes the available questionnaires per center. In order to compute the treatment outcome, the scores at admission and after treatment are used. As a consequence, the shared questionnaire (in this case, TQ) has to be available at both time points (*t*_0_ and *t*_1_) in order to learn a model that predicts the treatment outcome.

**Table 2:** Questionnaire categories and the available questionnaire data per center.

**Category**	**Questionnaire**	**CHA**	**UHREG**	**Citation**
Tinnitus distress	TQ	✓	✓	Goebel and Fichter, 2005
	TLQ	✓		Goebel and Hiller, 1998
	THI		✓	Jacobson and Newman, 1990
	TFI		✓	Meikle et al., 2012; Henry et al., 2016
	TBF12		✓	Greimel et al., 1999
	CGI		✓	Zeman et al., 2011
Physical strain	BI	✓		Brähler and Scheer, 1979
Depressivity	ADSL	✓		Hautzinger and Bailer, 1993
	BSF	✓		Hoerhold et al., 1993
	MDI		✓	Gislén et al., 2003
Stress	PSQ	✓		Levenstein et al., 1993; Fliege et al., 2005
Quality of life	SF8	✓		Beierlein et al., 2012
Coping	SWOP	✓		Scholler et al., 1999
Socio-demographics	SOZK	✓		
	[age, gender]	✓	✓	

*TQ, tinnitus questionnaire; TL, tinnitus localization and quality questionnaire; THI, tinnitus handicap inventory; TFI, tinnitus functional index; TBF12, tinnitus impairment questionnaire; CGI, clinical global impression; BI, Berlin complaint inventory; ADSL, general depression scalelong form; BSF, Berlin mood questionnaire; MDI, major depression inventory; PSQ, perceived stress questionnaire; SF8, short-form 8 health survey; SWOP, self-efficacy- optimism-pessimism scale; SOZK, socio-demographics questionnaire*.

## 3. Methods

### 3.1. Our Approach to Answer RQ1

To juxtapose the centers to each other and to the general population, we combine statistical testing and visualization tools.

According to RQ1, we compare the age distributions of our two samples *s*_1_ and *s*_2_ (one per center) for each gender separately and correct it for multiple testing over the 6 comparisons that are performed, using Bonferroni correction. In particular, we apply the Shapiro-Wilk test (Shapiro and Wilk, 1965) to check whether the samples follow a normal distribution: if yes, student's test (Gosset, 1908) is applied on the means of the samples; if not, MannWhitney U test (Neuhäuser, 2011) is conducted instead. All tests are performed with α = 0.05.

To visualize the distributions, we use kernel density estimation plots. In particular, we plot the two age distributions—of the German population and of the tinnitus patients from both clinical centers. For comparison purposes, we computed the percentage of people at a certain age for both data sets.

Secondly, we identified the regions of the age distributions that have similar shapes. I.e., we plot both distributions in a kernel density estimation plot and then interpret the zones where both distributions behave similarly—when there is a high ratio of German population for a certain age and a high percentage of tinnitus patients too.

This visual tool enables the identification of intervals of age in which both distributions show a high or low percentage of people. On the other hand, we may also identify age intervals in which one distribution has a high percentage of people and the other has lower values. The goal of this analysis is to identify age intervals in both populations (residents in Germany and tinnitus patients) where the percentage of people inside that interval is similar and where it differs. By identifying these differences and similarities, we explore the extent to which the two distributions agree. Concerning gender, the female and male ratio in Germany and in each clinical center are plotted using a bi-directional bar plot.

### 3.2. Our Approach to Answer RQ2

#### 3.2.1. Modeling Center Similarity With Respect to Questionnaire Score and Gender

A network-based analysis is performed to compute and interpret the similarity of tinnitus patients per gender and clinical center. In this network, nodes represent patients and edges represent the distance between them. The questionnaire score used to compute the edge weights is the TQ score. As a first step we standardize the questionnaire scores, as in Equation (1).


(1) $$\begin{array}{l} score_{p_{i,X}}^{\prime }=\frac{q_{p_{i,X}}-\mu_{q,X}}{\sigma_{q,X}} \end{array}$$

where *p*_*i,X*_ denotes a patient *i* from the clinical center *X*, *q*_*p*_*i,X*__ is the questionnaire score of patient *p*_*i,X*_, μ_*q,X*_ and σ_*q,X*_ are the mean and standard deviation of the questionnaire scores *q* in clinical center *X*, respectively.

The higher the score difference between patients, the weaker the connection between them. To account for that, a transformation 1/*x* is applied as shown in Equation (2), representing the edge weight.


(2) $$\begin{array}{l} w_{p_{i},p_{j},X}=\frac{1}{\left|score_{p_{i},X}^{\prime }-score_{p_{j},X}^{\prime }\right|} \end{array}$$

where *w*_*p*_*i*_, *p*_*j*_, *X*_ as the edge weight between patient *p*_*i*_ and *p*_*j*_ in clinical center *X*.

For the graph (or network) comparison, Tsitsulin et al. (2018) states that there are three main approaches: (i) direct methods, (ii) kernel methods, and (iii) statistical representations. Their approach falls in a different category, which is based on spectral representation. Tsitsulin et al. (2018) introduce the NetLSD (network laplacian spectral descriptor) method which creates a vector for each network using the “heat equation.” The difference between these vectors is computed and the final distance is the NetLSD metric. In comparison to the other approaches this method is able to meet three properties simultaneously: permutation invariance, scale-adaptivity, and size-invariance.

The permutation invariance property guarantees that the distance between two isomorphic graphs is equal to zero. The scale-adaptivity property is based on the representation including both local and global graph properties. Finally, the size-invariance property takes into account the magnitude of the graphs and can distinguish between graphs with comparable features but different magnitudes.

Datasets from different clinical centers vary in size and hence each graph that represents a different clinical center is of different size. The size-invariance property of NetLSD is critical in this work since it enables graph comparison between graphs of different sizes.

#### 3.2.2. Modeling Adherence

Since the centers use partially different questionnaires, we first organize the questionnaires into topical categories that reflect the (co-)morbidity they capture, independently of the questionnaire(s) in use. Then, we model *adherence with respect to a set of items I* for a patient *x*, denoted as *adh*_*I*(*x*) as the percentage of items from *I* answered by patient *x* over the total number of items in I, i.e., |*I*|. The set of items I can be a questionnaire or a topical category that encompasses more than one questionnaire. The adherence values are computed for each patient *x*, but in the following, we skip (*x*) from the notation for simplicity.

Let $A_{center}$ be the set of questionnaires in a center and $S=\cap_{center}A_{center}$ be the set of shared questionnaires between centers. Similarly, let $C_{center}$ be the set of categories in a center, where a category encompasses more than one questionnaire. Finally, let $V=\cap_{center}C_{center}$ be the set of topical categories common to all centers.

The setups—hereafter named as “adherence sets”—are subsets that represent different adherence rates expressed in percent.

On this basis, we generate sets of adherence features, as depicted on Table 3 and described hereafter. It is stressed that all sets refer to adherence at registration, i.e., at *t*_0_, since we intend to use these features to augment the original “basic set” of features used in the post-treatment predictors, i.e., at *t*_1_.

**Table 3:** Sets of adherence features over all questionnaires, shared questionnaires and categories.

**Name**	**Description**
Adherence set 1	average of the adherence values over all questionnaires of a center, i.e. $\frac{\underset{I\in A_{center}}{\sum }adh\text{\_}I}{\left|A_{center}\right|}$
Adherence set 2	set of distinct adherence values for each of the shared questionnaires, i.e. {adh_I|I∈S}
Adherence set 3	set of distinct adherence values of the questionnaires in all categories of a center, i.e. $\left\{adh\text{\_}I|I\in \cup_{c\in C_{center}}Q_{c}\right\}$, where *Q*_*c*_ is the set of questionnaires in category *c* of the center
Adherence set 4	set of distinct adherence values of the questionnaires of the shared categories, i.e. $\left\{adh\text{\_}I|I\in \cup_{c\in V}Q_{c}\right\}$, where *Q*_*c*_ is the set of questionnaires in the shared category *c*

“Adherence set 1” is the average adherence behavior in percent over all questionnaires at *t*_0_. I.e., for each patient, all the adherence rates for each questionnaire at *t*_0_ are calculated as the answered questions divided by the total number of questions of the respective questionnaire. This information serves as foundation to calculate the average adherence rate by summing up the percentages and divide this sum by the number of rates. This is a rough summary of the adherence behavior of each patient and can be compared among the centers ignoring, for, e.g., different sets of questionnaires. Since the current task is a prediction task at *t*_1_, the subset must be limited to the visit at *t*_0_. Otherwise, the train set would contain information regarding the target variable (post-treatment data).

“Adherence set 2” includes the average adherence rates for each questionnaire calculated by the answer behavior of a patient at *t*_0_. It should be noted, however, that in this subset only the adherence rates of the questionnaires that are common in both centers to be compared can be used. The rest are excluded from this subset. The reason is on the one hand the availability of the questionnaire and on the other hand the feasibility of the comparison for the cross-center prediction.

“Adherence set 3” assigns all the available questionnaires at a center to a category. For instance, tinnitus distress (TD) groups all tinnitus-related questionnaires. The adherence rate is calculated as the average of the questionnaire adherence rates per category. This is also exclusively for *t*_0_. In order to enable cross-center prediction, only categories available in both centers are included.

“Adherence set 4” is the average of the categories of subset 3 after removing the categories that have no counterpart in the opposite center.

### 3.3. Our Approach to Answer RQ3

To understand the relationship between the pre-treatment data and the post-treatment data within centers, a regressor is trained in the data of each center with the target being the post-data treatment of interest. In our case, this refers to the TQ score at *t*_1_.

In order to extend the comprehension of the similarity between patients across centers, we fit a regression model in one center and use it to predict the score after treatment of the patients from the other center. Aside from the previously stated adherence features, we introduce a new drill-down criterion to the experiments: gender. As a result, we utilize the models trained using patients from the CHA center to predict post-treatment data of patients from the UHREG center, per gender.

The regression models used are the following: linear regressor (LR), lasso, ridge, and support vector regressor (SVR). When developing these predictors, data are divided into a test and train set, and a 10-fold cross validation is performed inside the train set to choose the best model and its hyperparameters (with a grid search approach). For evaluation, standard measures, such as MAE (mean absolute error), MSE (mean squared error), and *R*^2^ (explained variance) are used.

Firstly, we train regressor models to predict the TQ score after treatment (*TQ*_*t*_1__), per center and with different features. In total, 5 experiments are performed, per center. The first and the one with the least number of features included is constituted by age, gender, and *TQ*_*t*_0__—the basic set. Then, we add adherence features to this set. Our main goal was to understand which set of features were most predictive of post-treatment TQ score, for within-center and between-center predictions.

## 4. Results

### 4.1. Comparison of Age and Gender Distributions in Germany and in Each Clinical Center

#### 4.1.1. Age

Despite the fact that demographics variables were analysed previously in tinnitus research (Seydel et al., 2013; Niemann et al., 2020), it was not yet explored that the age distribution may be influenced by another variable—for instance, the fact that we have less people with 70+ years in the population (due to the fact that life expectancy is within this range in Germany).

Hence, we propose to analyse the age distribution of tinnitus patients from the clinical centers along with the age distribution in Germany. Statistics from the German population age were gathered from a public data source, namely the German Federal Statistical Office (“Statistisches Bundesamt”)^1^.

**Figure 1:**
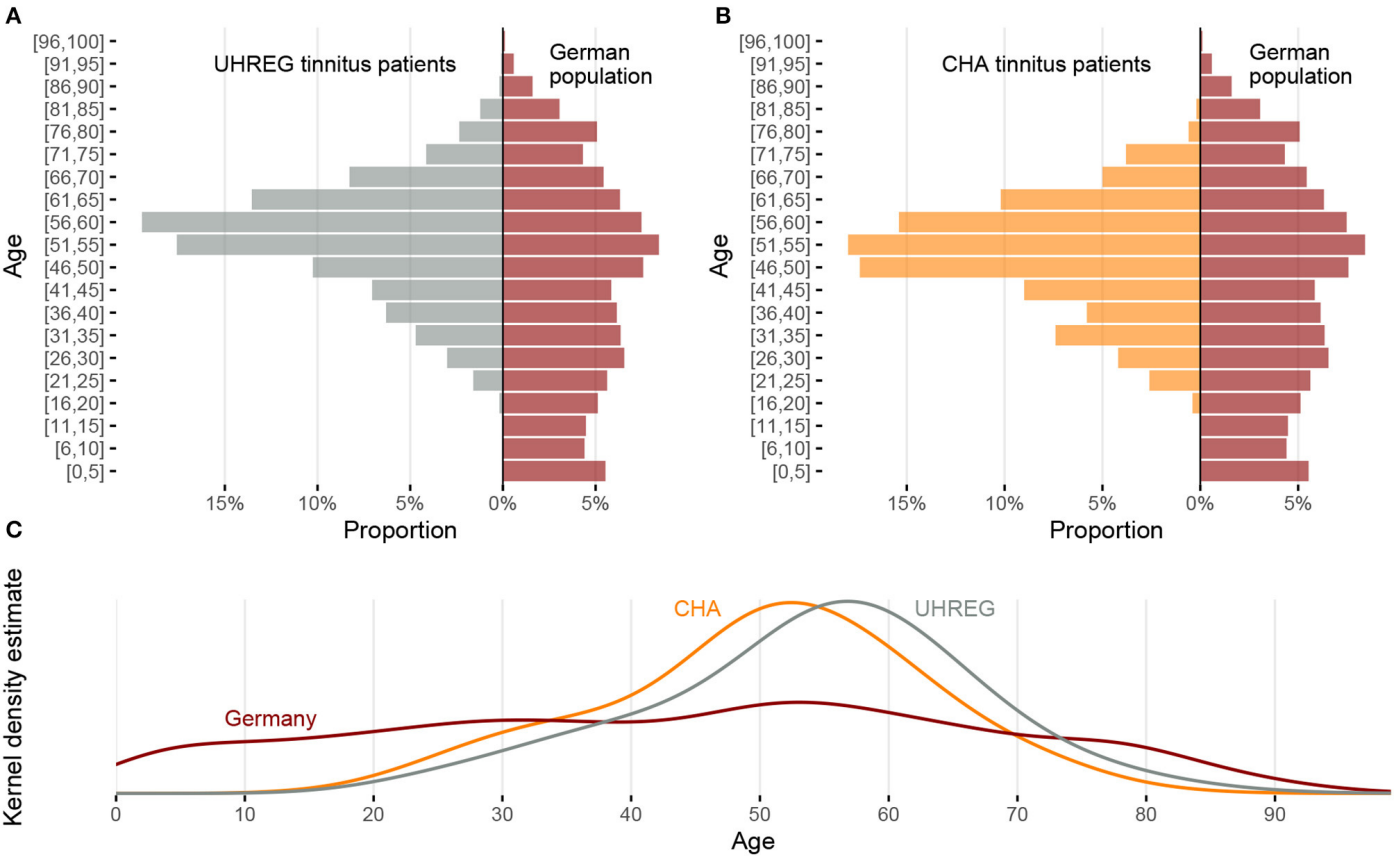
Comparison of age distribution between the two clinical centers and the German population. **(A)** Relative frequency by age intervals for UHREG (left) and the German population (right). **(B)** Relative frequency by age intervals for CHA (left) and the German population (right). **(C)** Density distributions of age for UHREG, CHA and the German population.

Figure 1 shows the distribution of age in Germany and in both clinical centers and the kernel density estimate of the age distributions.

In the histograms of Figures 1A,B and in the kernel density plots of Figure 1C, we see that the centers of all three distributions are between 50 and 60 years but the distributions are very different. The curves of the two clinics cross the curve of the German population in the interval between 30 and 40 years of age and a little earlier than 80 years of age.

**Figure 2:**
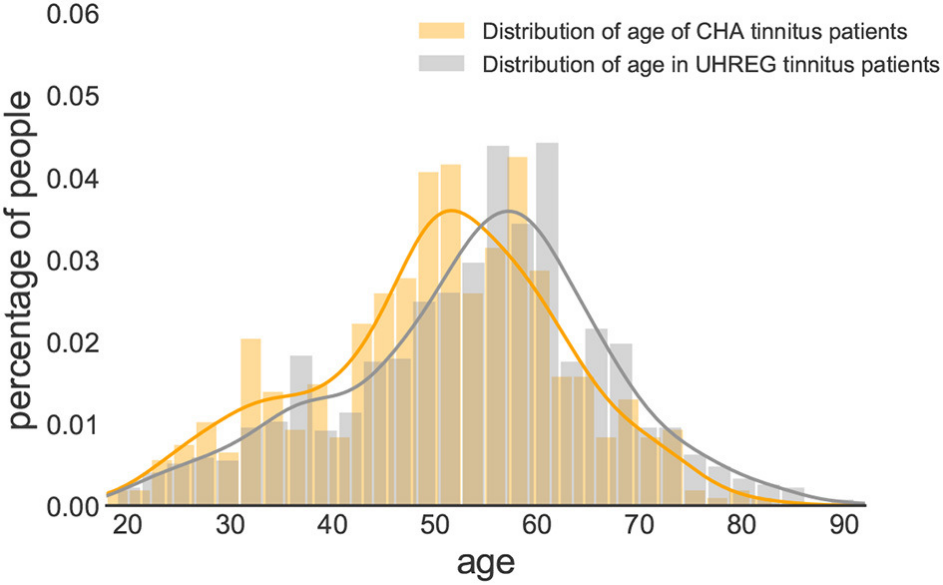
Age distribution comparison between clinical centers.

**Figure 3:**
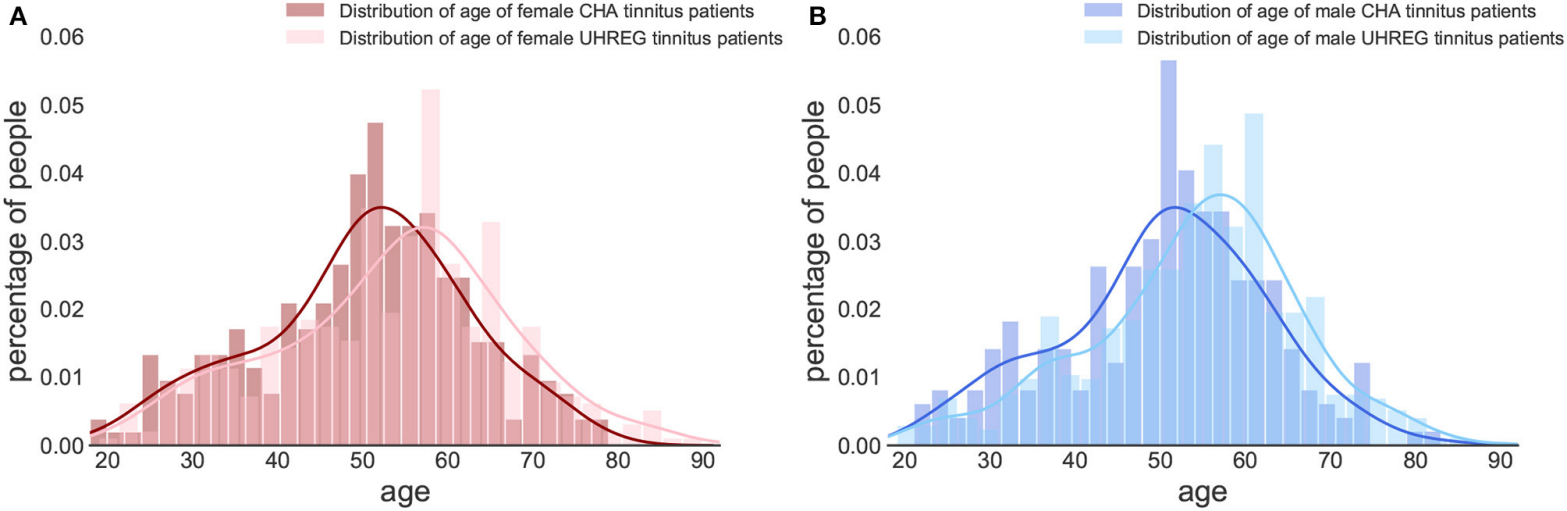
Age distribution per gender and clinical center. **(A)** Female tinnitus patients. **(B)** Male tinnitus patients.

Figures 2, 3 show the distribution of age per center and gender, respectively. The descriptive statistics of these distributions are shown in Table 4, per center and per gender.

**Table 4:** Descriptive statistics of age distributions.

**Data**	**N**	**min**	**max**	**mean**	**SD (σ)**	***p*-value (Shapiro)**
*age*_*uhreg*_	1,087	19	91	53.7	12.9	3.796*10^−8^
*age*_*cha*_	500	18	83	50.3	12.2	0.001*10^−1^
*age*_*uhreg,f*_	397	19	90	53.5	13.8	0.001
*age*_*uhreg,m*_	690	19	91	53.9	12.5	2.080*10^−6^
*age*_*cha,f*_	260	18	79	50.3	12.4	0.006
*age*_*cha,m*_	240	21	83	50.4	12.1	0.021

*SD, standard deviation; f, female; m, male; N, number of data points*.

We perform statistical tests to compare the age distributions between centers and determine whether there is statistical evidence to confirm that they are different. Given that the hypotheses of normality (Shapiro Wilk test) are rejected for all subsets in Table 4, we elect the Mann-Whitney test to compare location measures between samples. In particular, we test (1) whether female (first line of Table 5) vs. male tinnitus patients (second line) featured similar age distributions in the two centres and (2) whether female and male patients featured similar age distributions within each centre (3rd line for UHREG, 4th line for CHA) and, finally, whether female tinnitus patients of one centre featured similar age distributions as male patients in the other center (last two lines).

**Table 5:** Medians, Mann-Whitney U-statistic and *p*-value of a Mann-Whitney two-sided test for comparison of two samples.

***Sample 1 (s*** **_1_)**	***Sample 2 (s*** **_2_)**	***Median (s*** **_1_)**	***Median (s*** **_2_)**	**U (statistic)**	**p-value**
*age*_*uhreg,f*_	*age*_*cha,f*_	55	51	59233.0	<0.01^*^
*age*_*uhreg,m*_	*age*_*cha, m*_	55	51	97328.5	<0.01^*^
*age*_*uhreg,m*_	*age*_*uhreg,f*_	55	55	138974.5	0.69
*age*_*cha,m*_	*age*_*cha,f*_	51	51	31163.0	0.98
*age*_*uhreg,m*_	*age*_*cha,f*_	55	51	105214.0	<0.01^*^
*age*_*uhreg,f*_	*age*_*cha,m*_	55	51	54711.5	<0.01^*^

An asterisk * indicates statistical significance after Bonferroni correction of the critical value. Therefore, the p_crit_ = α/n_comparisons_ = 0.05/6 ≈ 0.008 . age_uhreg,f_ denote the age of female tinnitus patients in UHREG, age_cha,f_ the age of female tinnitus patients in CHA, age_uhreg,m_ denotes the age of male tinnitus patients in UHREG and age_cha,m_ the age of male tinnitus patients in CHA.

As can be seen in the table, it can be stated that female and male patients within the same center follow the same age distribution (*H*_0_ cannot be rejected). The other null hypotheses are rejected. Based on these findings and complementing it with the analysis of the histograms, we can infer that the age distribution in UHREG is consistently higher than the one in CHA, regardless of gender.

#### 4.1.2. Gender

**Figure 4:**
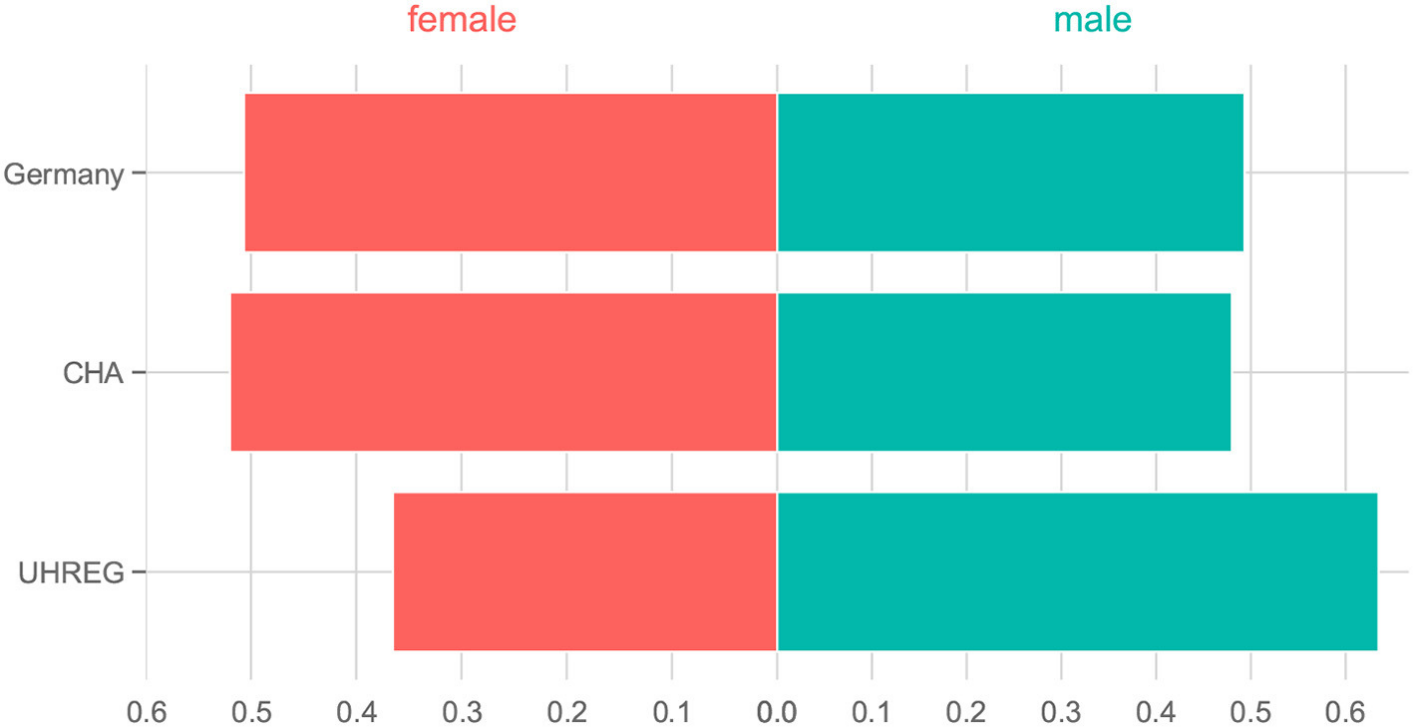
Percentage of people per gender in Germany and in each clinical center.

Regarding gender, Figure 4 shows the distributions of gender in Germany [from the 'Statistisches Bundesamt' (see text footnote 1)] and in both clinical centers. We can see that distribution of gender in CHA is close to the one in Germany. In contrast, UHREG has a higher percentage of male individuals than the general German population.

### 4.2. Clinical Center Similarity With Respect to TQ Score Per Gender

A network-based analysis is carried as a method to capture the similarity between patients, with respect to the TQ score. Four networks are generated: one per gender and one per center.

**Figure 5:**
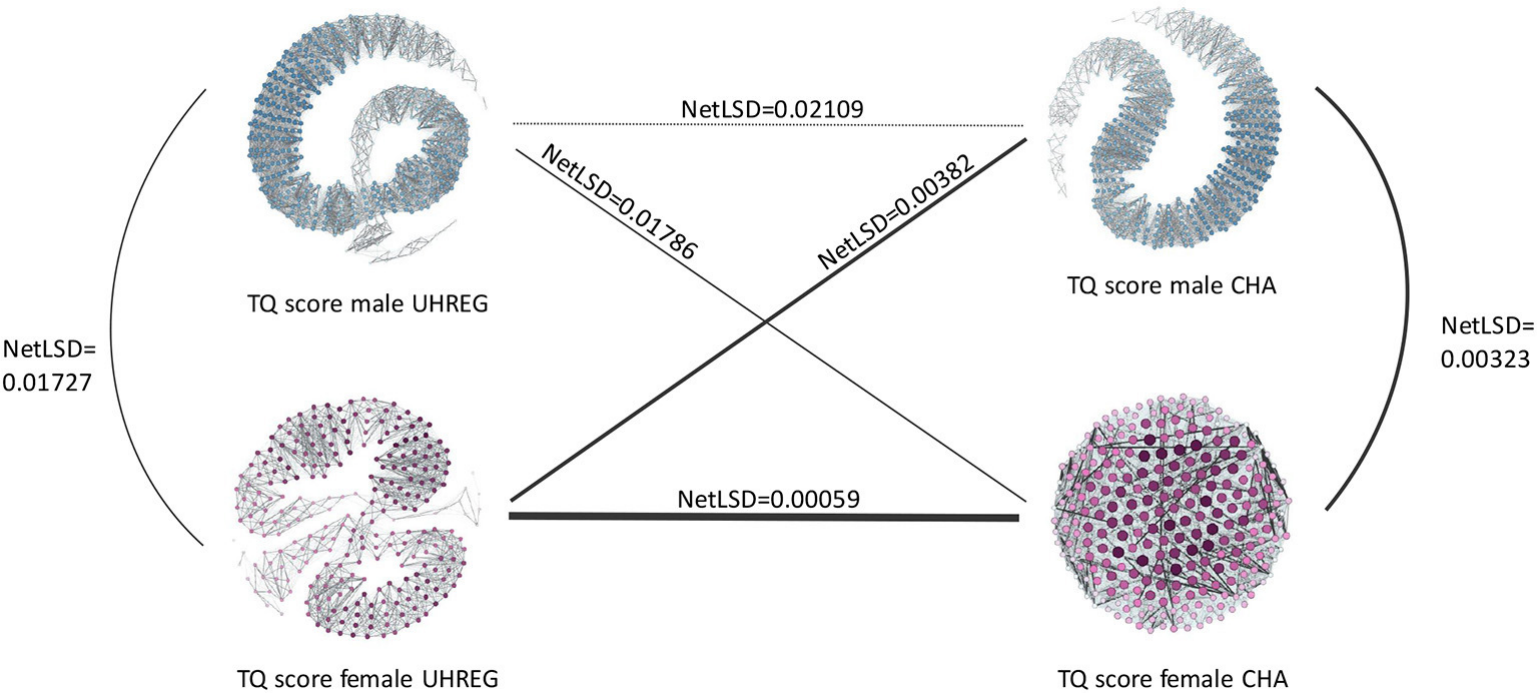
NetLSD distances of graphs with *TQ*_*t*_0__ per clinical center and gender.

The four networks that represent distinct groups of tinnitus patients are illustrated in Figure 5. The purple networks represent TQ score data from female patients, whereas the blue networks represent TQ score data from male patients. In this analysis, the emphasis is on the TQ scores and similarity is modeled with respect to this variable.

As previously stated, the nodes represent the patients, while the edges connecting them show the similarity between them. Denser areas in each network reflect patients who are similar to one another with respect to their TQ score, whereas darker and thicker edges indicate a stronger connection between patients and therefore a high similarity.

The NetLSD score provides a measure of the distance between networks. The lower this score, the higher similarity between the networks. Given the purpose of this paper, we focus on the difference of the distance values only: Figure 5 illustrates that, compared to the respective other networks, female patient networks in UHREG and CHA are the most similar, and male patient networks in UHREG and CHA the most dissimilar with respect to the TQ score.

Irrespective of the findings in Table 5, which demonstrated statistically significant differences in the age distributions of female patients within centres, the female patients' networks were the most similar according to the TQ score. By contrast, male tinnitus patients, who also featured significant differences in the age distributions, differed most in terms of their TQ scores.

The blank regions of the graphs indicate that there is no edge linking nodes on opposite sides of the graph. This occurs following the graph pruning phase, during which non-statistically significant edges are deleted. These empty areas differ amongst graphs due to their diverse characteristics; some have more statistically significant edges than others and hence are more connected.

### 4.3. Adherence Feature Sets

In order to capture the diverse spectrum of patient behavior in each center, all available and applicable records are used to get the adherence meta-information. Table 2 summarizes the available questionnaires per center. This is a necessary step before computing the adherence sets, since a common feature space (in this case, pre-treatment data) is required to compute some of the sets.

The basic set of features is considered to be the common pre-treatment data between centers. In the case of the current study, this corresponds to age, gender and *TQ*_*t*_0__. For both centers the meta-information is calculated. “Adherence set 1” includes for both CHA and UHREG just a single feature with an average adherence rate. “Adherence set 2” includes the common questionnaires.

The only shared questionnaire between CHA and UHREG is the TQ. Therefore, this subset includes only one feature: the average adherence rate for TQ at *t*_0_. “Adherence set 3” focus on the average adherence rate of common categories. There are two shared categories: tinnitus distress (TD) and depressivity (D), although both centers, except for the TQ, have different sets of questionnaires per category. “Adherence set 4” is the average of both common categories, which means in fact the average of the underlying questionnaires at *t*_0_.

Table 6 shows the average and the standard deviation (SD) of the adherence features of each center. One important remark is that there are some patients with missing values for the gender from the clinical center CHA. This is also shown in Table 6 and is of importance for the prediction task.

**Table 6:** Average and SD of adherence features.

**Center**	**Adherence set**	**Gender**			

		**f**	**m**	**NA**	**all**
CHA	Adherence set 1	99.2% ± 5.3%	99.2% ± 5.5%	55.9% ± 24.6%	97.7% ± 10.5%
	Adherence set 2	100.0% ± 0.0%	100.0% ± 0.0%	14.3% ± 35.9%	97.0% ± 17.0%
	Adherence set 3	TD: 99.8% ± 2.9% D: 98.8% ± 7.7%	TD: 99.8% ± 2.9% D: 98.9% ± 8.2%	TD: 50.0% ± 22.4% D: 76.2% ± 25.6%	TD: 98.1% ± 10.4% D: 98.1% ± 10%
	Adherence set 4	99.3% ± 4.6%	99.4% ± 5.2%	63.1% ± 20.3%	98.1% ± 9%
UHREG	Adherence set 1	66.7% ± 21.4%	68.5% ± 19.9%		67.9% ± 20.3%
	Adherence set 2	100.0% ± 0.0%	99.9% ± 0.3%		99.9% ± 0.2%
	Adherence set 3	TD: 62.5% ± 19.8% D: 87.5% ± 33.8%	TD: 65.2% ± 17.6% D: 84.8% ± 36.3%		TD: 64.3% ± 18.3% D: 85.7% ± 35.2%
	Adherence set 4	75.0% ± 25.7%	75.0% ± 25.9%		75.0% ± 25.6%

*TD, tinnitus distress; D, depressivity; f, female; m, male*.

### 4.4. Prediction of Questionnaire Score After Treatment

Table 7 shows the predictions of the *TQ*_*t*_1__ per center, using different feature sets. These sets differ in their features. The age, gender, and *TQ*_*t*_0__ are denoted as basic set of features. Then, four adherence feature sets are added - sets 1, 2, 3 and 4. The target variable is set to be the TQ score at *t*_1_, which we refer to as *TQ*_*t*_1__. Patients with missing values for the used features (for example, missing values of gender in CHA as reported in Table 6) are excluded from this study.

**Table 7:** Prediction of TQ score at *t*_1_ with and without adherence feature sets.

**Center**	**Metric**	**Features**	**LR**	**LASSO**	**Ridge**	**SVR**
UHREG	MAE	Basic set	8.896	13.3	9.4	13.5
	MSE	(*N* = 70)	123.4	228.2	157.3	273.3
	*R* ^2^		0.674	0.585	0.392	0.481
	MAE	Basic set + adherence set 1	9.426	10.490	**9.0**	12.0
	MSE	(*N* = 70)	165.148	199.0	**117.7**	227.4
	*R* ^2^		0.495	0.349	**0.714**	0.431
	MAE	Basic set + adherence set 2	12.9	8.3	11.5	10.0
	MSE	(*N* = 70)	234.6	114.2	193.8	170.1
	*R* ^2^		0.443	0.646	0.391	0.545
	MAE	Basic set + adherence set 3	11.9	9.3	8.9	10.6
	MSE	(*N* = 70)	233.5	115.8	144.7	199.8
	*R* ^2^		0.180	0.683	0.585	0.299
	MAE	Basic set + adherence set 4	11.2	8.5	8.6	8.6
	MSE	(*N* = 70)	187.6	146.6	132.9	116.2
	*R* ^2^		0.406	0.527	0.607	0.688
CHA	MAE	Basic set	6.220	6.680	6.955	6.760
	MSE	(*N* = 500)	62.388	76.331	79.239	80.148
	*R* ^2^		0.804	0.742	0.766	0.720
	MAE	Basic set + adherence set 1	6.3	7.8	6.9	7.4
	MSE	(*N* = 218)	65.5	99.2	86.8	89.3
	*R* ^2^		0.829	0.745	0.771	0.780
	MAE	Basic set + adherence set 2	7.1	8.2	6.8	5.7
	MSE	(*N* = 218)	85.9	104.1	77.3	54.2
	*R* ^2^		0.761	0.743	0.692	0.823
	MAE	Basic set + adherence set 3	7.3	6.8	7.3	6.3
	MSE	(*N* = 218)	78.6	73.6	82.2	71.5
	*R* ^2^		0.702	0.771	0.817	0.729
	MAE	Basic set + adherence set 4	7.2	7.0	6.8	**6.8**
	MSE	(*N* = 218)	90.4	78.7	70.8	**73.2**
	*R* ^2^		0.749	0.761	0.810	**0.839**

*The error metrics presented are as follows: MAE (mean absolute error); MSE (mean squared error); R^2^ (explained variance) and the regressors trained are as follows: LR (linear regressor), LASSO, Ridge and SVR (support vector regressor). In bold are the results of the regressor trained with the set of features that achieved the highest R^2^, per center*.

For UHREG, the ridge regressor achieves the best results using the basic feature set and and the “adherence feature set 1”. For CHA, the SVR (support vector regressor) regressor achieves the best results using the basic feature and “adherence set 4”.

To predict UHREG patients' treatment outcome, three models are trained with three different datasets: (i) all CHA patients, (ii) female CHA patients only and (iii) male CHA patients only. These models are then tested on UHREG patient data. Table 8 illustrates the results.

**Table 8:** Prediction of *TQ* at *t*_1_ on UHREG tinnitus patients with model trained on CHA tinnitus patients (results from the model with the highest *R*^2^ are shown).

**Model trained on**	**Predicted on**	**Adherence**	**MAE**	**MSE**	***R*** **^2^**
CHA all	UHREG all (*N* = 70)	Basic set	9.9	158.7	0.514
(*N* = 500)	**UHREG female (*****N*** **=** **24)**		**9.7**	**154.1**	**0.533**
	UHREG male (*N* = 46)		10.0	161.0	0.500
	UHREG all (*N* = 70)	Basic set + adherence set 1	9.9	160.5	0.510
	**UHREG female (*****N*** **=** **24)**		**9.6**	**153.8**	**0.532**
	UHREG male (*N* = 46)		10.1	164.0	0.493
	UHREG all (*N* = 70)	Basic set + adherence set 2	10.0	160.4	0.517
	**UHREG female (*****N*** **=** **24)**		**9.7**	**153.5**	**0.535**
	UHREG male (*N* = 46)		10.1	164.0	0.503
	UHREG all (*N* = 70)	Basic set + adherence set 3	10.0	162.0	0.505
	**UHREG female (*****N*** **=** **24)**		**9.7**	**154.8**	**0.529**
	UHREG male (*N* = 46)		10.2	165.9	0.487
	UHREG all (*N* = 70)	Basic set + adherence set 4	10.1	162.9	0.505
	**UHREG female (*****N*** **=** **24)**		**9.8**	**155.6**	**0.530**
	UHREG male (*N* = 46)		10.2	166.7	0.487
CHA female	UHREG female (*N* = 24)	Basic set	**9.4**	**148.8**	**0.544**
(*N* = 260)		Basic set + adherence set 1	9.5	157.0	0.519
		Basic set + adherence set 2	9.8	153.9	0.535
		Basic set + adherence set 3	11.0	217.2	0.420
		Basic set + adherence set 4	9.9	167.1	0.492
CHA male	UHREG male (*N* = 46)	Basic set	10.2	166.8	0.490
(*N* = 240)		Basic set + adherence set 1	10.1	163.1	0.496
		Basic set + adherence set 2	10.1	162.5	0.496
		Basic set + adherence set 3	**9.8**	**161.5**	**0.498**
		Basic set + adherence set 4	10.1	164.6	0.489

*In bold are the results from the results predicted in the subset and the features that achieved the highest R^2^*.

We can conclude that the female tinnitus patients from UHREG are the most predictable among all learners from CHA trained with all their patients. The basic set and the “adherence 2” set of features are the most successful in terms of *R*^2^.

In the second part of Table 8, only female patients from CHA are used to train a model and this model is used to predict the female patients from UHREG. In this example, the basic feature set provides the highest *R*^2^.

Finally, a learner is trained only on the male patients from CHA and used to predict the *TQ*_*t*_1__ on patients from UHREG. The best set of features is the basic set with the “adherence set 3” set of features. This means that these features were the ones that best predicted *TQ*_*t*_1__ on patients from UHREG and can thus be deemed the most useful for understanding the post-treatment TQ score (in comparison to the other set of features).

## 5. Discussion

In tinnitus research, age and gender have been two variables of interest to analyse. Recently, Niemann et al. (2020) showed that women present a higher level of depression and tinnitus-related distress. Another study by Seydel et al. (2013) recognized age and gender as the most relevant factors to predict tinnitus distress. Rodrigo et al. (2021) investigated the impact of several features for the success of internet-based CBT (cognitive behavioral therapy) on tinnitus patients. In this study, age and gender were used as features but the feature that proved the highest impact on the outcome of the treatment was the education level of the patients. Their findings indicated that patients with a higher level of education were more likely to succeed after treatment.

In the present study, we found that the age distribution of the general German population is partly reflected in the age distributions of the two centers' tinnitus patients. All three distributions reflect a drop in the age window 75+. An explanation could be that elderly patients are less mobile, especially those in rural areas. Another explanation could be that the likelihood of other conditions increases with age - which might be associated with a perception of tinnitus as relatively less distressing. These are hypotheses and more investigation is needed to understand this pattern of chronic elderly tinnitus patients. Middle-aged people, on the other hand, were considerably over-represented in the tinnitus centers relative to the general German population. Therefore, the percentage of patients that seek medical care for tinnitus within middle-aged people is higher than for the other age ranges and it cannot be explained by the German population characteristics.

Within, but not between centers, same-gender patients were found to differ significantly in age. While this is true for age, the results from the netLSD distances show that female patients from different centers are, among all pairs of networks, the most similar according to their TQ score. Another finding was the fact that age of female and male patients from different centers were not statistically different.

Another intriguing finding was that models trained with adherence features outperformed baseline models that did not include these features. The model trained with female CHA tinnitus patients was more predictive of female UHREG patients than the model trained with male CHA patients and tested on male UHREG patients. As a result, UHREG female patients are better predicted with CHA data than male patients. This means that treatment-improvement rates and patterns improve more similarly in females than males, when centers are compared. This result can be cross-checked with the NetLSD distance results. In this analysis, female tinnitus patients from different centers were also found to be more similar than male patients with respect to questionnaire score at *t*_0_. Hence, we can conclude that both results from different methods agree in terms of similarity. Despite the heterogeneity of clinical centers and the fact that these are preliminary results, there may be an indication that patients across clinical centers share similar characteristics. It is worth noting that the amount of available data for analysis varies between clinical centers, which may have an impact on how representative they are of their patients. As a result, we consider the reported findings to be preliminary and the analysis should be expanded to larger datasets.

The adherence features that summarize the information the most, “adherence 1” and “adherence 4,” produced the greatest improvement in the *R*^2^ value in the gender-agnostic intra-center analysis. In the inter-center prediction using all the data from the training center, however, “adherence 2” outperforms the other adherence sets. Nonetheless, the “basic” set outperforms the other 3 adherence features for all, as well as for female and male. Thus, in the cross-center prediction scenario, information on adherence in the common questionnaire(s) seems to be a useful addition.

## 6. Conclusion

In this article, we performed various analyses to compare tinnitus patients from two different large clinical centers. These comparisons were carried mainly focusing on the questionnaire scores before and after treatment, socio-demographics and adherence to the filling of the questionnaires. For that, visualization and prediction methods were implemented along with a network-based representation of the data.

The main findings can be organized into three. The first one being that the distribution of age in Germany agrees, in some age ranges, to the ones from the tinnitus patients from both centers. This indicates good and representative reach of the specialist treatment centers in offering care for their target populations. The second, that female tinnitus patients from one center (CHA) are more predictive of the female patients of the other center (UHREG) than male patients. This result is complemented by the fact that our network-based approach to compute the similarity between networks also agreed that female tinnitus patients were more similar across centers than male patients, with respect to treatment score at *t*_0_. The third, that including meta-information about the adherence of the patients to the filling of the questionnaires improved the baseline predictions of post-treatment data.

The evaluation's findings could be supported further by finding datasets with more overlapping questionnaires but also closer in terms of sample size. Finding such datasets will be a future task. Incorporating information about gender and adherence could improve the prediction task. Future research should place these findings to the test by applying intra- and inter-center predictions to other centers.

## Data Availability Statement

The data analyzed in this study is subject to the following licenses/restrictions: The datasets for this article are not publicly available because no consent of the patients to publish their data was obtained. Requests to access these datasets should be directed to BM, birgit.mazurek@charite.de for the CHA dataset and WS, winfried.schlee@gmail.com for the UHREG dataset.

## Ethics Statement

The studies involving human participants were reviewed and approved by Ethics Committee of the University Medicine Charité Berlin for the CHA Dataset and the Ethics Committee of the University Regensburg for the UHREG Dataset. The patients/participants provided their written informed consent to participate in this study.

## Author Contributions

CP and MSc designed and performed the data analysis and wrote the original draft. CP designed and performed the network-based analysis. MSc modeled adherence. CP and UN designed the visualizations. UN optimized the visualizations on interpretability. VU supported on questionnaire interpretation and data preparation. BB, PB, and BM curated the dataset with patients admitted to the Tinnitus Center, Charité – Universitätsmedizin Berlin. JS, BL, and WS worked on data curation and model interpretation. BB, PB, BM, WS, and MSp supervised the data analysis and reviewed and edited the manuscript. BM and MSp led the project. All authors contributed to the article and approved the submitted version.

## Funding

Part of this project has received funding from the European Union's Horizon 2020 Research and Innovation Programme under grant agreement number 848261.

## Conflict of Interest

The authors declare that the research was conducted in the absence of any commercial or financial relationships that could be construed as a potential conflict of interest.

## Publisher's Note

All claims expressed in this article are solely those of the authors and do not necessarily represent those of their affiliated organizations, or those of the publisher, the editors and the reviewers. Any product that may be evaluated in this article, or claim that may be made by its manufacturer, is not guaranteed or endorsed by the publisher.

## Footnotes

^1^https://www.destatis.de, site accessed in March 2020.
